# St. Bartholomew's Hospital

**Published:** 1903-03-21

**Authors:** 


### EDITOR'S LETTER-BOX.

[Our Correspondents are reminded that prolixity is a great bar to publication, and that brevity of style and conciseness of statement greatly facilitate early insertion.]

### ST. BARTHOLOMEW'S HOSPITAL.

A "BART.'S MAN" writes: There is a letter in the St. Bartholomew's Hospital *Journal* of this month from a correspondent—an old Bart.'s man—who complains that, when he is asked his opinion of the suggested appeal for charitable contributions on the part of St. Bartholomew's Hospital, he finds it very difficult to give outsiders an opinion which he is able to back up by an appeal to the facts. He therefore suggests that some data may be given whereby Bart.'s men may answer the rather unfriendly criticisms that at present they have frequently to listen to without being able to answer. It is exactly these data that the public, charitable and otherwise, are so anxious to be put in possession of, and it is with some feeling of hope that one turns to the editorial reply. The editor of St. Bartholomew's Hospital *Journal* is a modest man, and a kind-hearted one withal, and consequently it is with sorrow that he informs the world at large that, with the single exception of himself, editors are not overfond of accuracy, and it is his pious hope that, again with the exception of himself, of course, a small but warm corner may be reserved for them between Ananias and the little boys who mocked Elisha. We understand that the editors of the *Times* and other daily papers are the little boys, and that he himself represents Elisha; or is he the destroying bear? Doubtless an excessive devotion to accuracy is an admirable thing in some ways, but it evidently is not an infallible preventative against making mistakes; frail humanity will err, and immediately below the exposition of certain vague and not very accurate figures and statements which are supposed to finally dispose of those who advocate the removal of St. Bartholomew's Hospital from its present site—(to the initiated we have only to mention that the editorial reply states that the area of land purchased from Christ's Hospital measured one acre and a half—to be out in one's figure by about £40,000 or £50,000 is, after all, a mere bagatelle to the infallible)—the editor in another note apologises for no fewer than ten mistakes which he allowed to pass in a single article published in the previous issue of the journal. It must have been so very easy for a man who suffers from over-accuracy to let "Columbus" pass for "calculus," and "salcormen" for "salernum." Further in the course of his dissertation on the "Barts." question he sadly deprecates the fact that editors, with the exception of himself, do not abstain from making personal attacks, though he, quite inadvertently of course, informs us that the chairman of a certain great London hospital, which he names, is one of those who think that everything is fair in love, war and charity. Such a personal accusation as this is of course merely a slip of the pen, which might occur even to the most accurate of men. On the whole, however, we think the writers of the many letters of inquiry had better wait till the next issue of the magazine before using the information in the current number, as the Editor will then have had the time he seems to require for correcting his articles.

